# Digital Interventions to Promote Healthy Eating in Children: Umbrella Review

**DOI:** 10.2196/30160

**Published:** 2021-11-25

**Authors:** Rachel Prowse, Sarah Carsley

**Affiliations:** 1 Division of Community Health and Humanities Faculty of Medicine Memorial University of Newfoundland St. John's, NL Canada; 2 Health Promotion, Chronic Disease and Injury Prevention Public Health Ontario Toronto, ON Canada

**Keywords:** children, healthy eating, eHealth, nutrition intervention, nutrition education, food literacy, digital health, virtual delivery, digital interventions, nutrition interventions, best practices, education, mobile phone

## Abstract

**Background:**

eHealth and web-based service delivery have become increasingly common during the COVID-19 pandemic. Digital interventions may be highly appealing to young people; however, their effectiveness compared with that of the usual face-to-face interventions is unknown. As nutrition interventions merge with the digital world, there is a need to determine the best practices for digital interventions for children.

**Objective:**

The aim of this study is to examine the effectiveness of digital nutrition interventions for children on dietary outcomes compared with status quo interventions (eg, conventional face-to-face programming or nondigital support).

**Methods:**

We conducted an umbrella review of systematic reviews of studies assessing primary research on digital interventions aimed at improving food and nutrition outcomes for children aged <18 years compared with conventional nutrition education were eligible for inclusion.

**Results:**

In total, 11 systematic reviews published since 2015 were included (7/11, 64%, were of moderate quality). Digital interventions ranged from internet, computer, or mobile interventions to websites, programs, apps, email, videos, CD-ROMs, games, telehealth, SMS text messages, and social media, or a combination thereof. The dose and duration of the interventions varied widely (single to multiple exposures; 1-60 minutes). Many studies have been informed by theory or used behavior change techniques (eg, feedback, goal-setting, and tailoring). The effect of digital nutrition interventions for children on dietary outcomes is small and inconsistent. Digital interventions seemed to be the most promising for improving fruit and vegetable intake compared with other nutrition outcomes; however, reviews have found mixed results.

**Conclusions:**

Owing to the heterogeneity and duration of digital interventions, follow-up evaluations, comparison groups, and outcomes measured, the effectiveness of these interventions remains unclear. High-quality evidence with common definitions for digital intervention types evaluated with validated measures is needed to improve the state of evidence, to inform policy and program decisions for health promotion in children. Now is the time for critical, robust evaluation of the adopted digital interventions during and after the COVID-19 pandemic to establish best practices for nutrition interventions for children.

## Introduction

### Background

Poor nutrition is a leading risk factor for noncommunicable diseases, such as cardiovascular disease, cancer, stroke, and diabetes [1]. Dietary risks (eg, diets low in fruits, vegetables, whole grains and high in red and processed meat, and sugar-sweetened beverages [SSBs]) are among the top 3 risk factors for global attributable deaths [2]. Proper child nutrition is foundational in preventing chronic disease later in life [3]. However, child wasting, underweight, and stunting remain among the top 10 leading contributors to disability-adjusted life years for children aged 0-9 years globally [2]; iron deficiency was the top risk factor of attributable disability-adjusted life years for individuals aged 10-24 years in 2019 [2].

Dietary intake is determined by a plethora of factors ranging from individual characteristics such as nutrition knowledge, self-efficacy, and income to societal factors such as food marketing and media, and supportive environments to access affordable healthy food [4-6]. Food literacy is an umbrella concept related to food skills and knowledge necessary to perform healthy eating behaviors and links individual-level attributes to the food environment in which eating behaviors take place [7]. As a determinant of diet, food literacy is a focus of nutrition interventions to improve individual and population diets.

Although face-to-face interventions are accepted, evidence-based approaches to deliver nutrition interventions [8] and the adoption of digital technologies, particularly during the COVID-19 pandemic, have required practitioners and policy makers to explore novel approaches to support healthy practices. The use of mobile apps by dietitians and their clients is emerging—57% of 117 dietitians surveyed in Canada used apps in their practice and 84% of those who did not use apps were interested in adopting them in the future [9]. A growing number of nutrition and diet apps are available on app stores (eg, Google Play), which provide unique features to users, such as self-monitoring, goal-setting, education, push notifications, message forums, personalized messages, and rewards, to promote healthy behavior change [10-13]. Credible on-demand nutrition information has previously been available for consumers and health professionals in Canada through websites, social media, apps, and telephone platforms. One web-based and telephone nutrition service in Canada yielded 1000 telephone calls, 1000-1500 email inquiries, and >240,000 website page views each month [14]. However, the effectiveness of digital interventions to improve diet and lifestyle, compared with conventional educational approaches, has not been well established [8,15,16].

As *digital natives*, today’s youth may find digital approaches to nutrition education more meaningful and impactful than the conventional approaches [17]. The internet, telehealth, gaming, social media, mobile apps, and wearable devices are few digital platforms that have been used to promote health among the youth, with varied impacts [18]. Before the COVID-19 pandemic, digital interventions were already rapidly developing as anonymous, accessible, and cost-effective interventions appealing to the youth [16]. During the pandemic, most health care, public health, and community services rapidly transitioned to the web, attempting to mimic traditional services through digital means. Digital technologies can improve equitable health service delivery; however, several knowledge gaps hinder the practitioners’ ability to optimize their use [19]. The opportunity for service providers to develop and implement evidence-based digital health care or health promotion interventions, including those who serve children and youth [20], must be met with evaluating the existing evidence to guide real-world decisions in real time.

### Objective

The primary aim of this review is to examine the effectiveness of digital nutrition interventions on food literacy outcomes in children (<18 years) compared with the status quo interventions (eg, face-to-face programming or nondigital support). Second, this review aims to explore the features of digital nutrition interventions that are most effective in promoting food literacy.

## Methods

We conducted an umbrella review of systematic reviews. This approach was used to synthesize high-level evidence to support health-related programs and policy decision-making [21]. Following recommended practices for umbrella reviews, we stated a clear objective informed by stakeholders; defined *systematic review*; specified relevant inclusion and exclusion criteria; structured our search strategy; and conducted dual screening, explicit data extraction, and quality appraisal [22].

### Search Strategy

A literature search was conducted in November 2020 by a librarian for articles published between 2015 and the search date. These year limits were used to minimize the inclusion of archaic digital *innovations*. Eight databases were searched (Ovid MEDLINE, PsycINFO, Global Health, CINAHL, SocINDEX, AgeLine, Child Development and Adolescent Studies, and Scopus) with the following search terms: digital interventions, telehealth, telemedicine, videoconferencing, social media, apps, health promotion, public health, preventive health services, diet, food, eating, nutrition, and breastfeeding. References from the included articles were hand searched for additional relevant reviews. A forward search of relevant review protocols was completed in December 2020 to include the published results. The full search strategy is available upon request.

### Study Inclusion and Exclusion Criteria

An a priori population-intervention-comparison-outcome statement [23] guided the inclusion and exclusion criteria: systematic reviews of studies of digital interventions aimed at improving food and nutrition outcomes for children <18 years compared with conventional nutrition education were eligible for inclusion.

#### Types of Participants

Reviews were included if they evaluated digital interventions aimed at children <18 years and reported separate results for children. Reviews that focused on interventions for children with a chronic disease, with the exception of overweight and obesity, were excluded.

#### Types of Interventions

Only digital interventions or interventions with both digital and nondigital (eg, print or face-to-face) components were included. An unrestricted definition of *digital* was used to obtain evidence that can increase the relevance of the umbrella review for decision-makers [21]. Interventions that used eHealth, mobile health (mHealth), telehealth (Textbox 1), or other electronic or internet-based programs, applications, or games where participants engaged through portable computers, desktop computers, mobile devices, and wearable devices were included. Reviews were excluded if they only reported on face-to-face interventions or aggregated results from face-to-face or print interventions with digital interventions.

**Definitions of eHealth, mobile health (mHealth), and telehealth.**

**Definitions**

- eHealth: “the use of information communications technology in support of health and health-related fields.” [24]
- mHealth: “an element of eHealth which focuses solely on mobile technology and is defined as ‘the use of mobile wireless technologies for public health’.” [24]
- Telehealth: “various types of health care when patient and provider are geographically separated—it can involve videoconferencing, telephone calls, electronic data transmission, and other ways of communicating over the Internet.” [25]

#### Comparators

We included reviews that compared digital interventions with no intervention, minor interventions (eg, wait list), nondigital nutrition interventions (eg, print), nonnutrition digital interventions (eg, physical activity website), and conventional face-to-face programming or usual education. It was not possible to restrict our analysis to only reviews with conventional face-to-face programming because the relevant systematic reviews included a wide range of controls and comparison types.

#### Types of Studies

Systematic reviews (including non-Cochrane reviews) and meta-analyses were included; narrative and scoping reviews were excluded. We defined *systematic reviews* as a review of evidence with clearly stated research questions, search strategy that is reproducible, inclusion and exclusion criteria, selection methods, quality and risk of bias assessment, and evidence synthesis [26]. Various study designs included in the systematic reviews were acceptable, including randomized controlled trials (RCTs), quasi-experiments, and cross-sectional studies, as these are common designs in nonclinical research. Reviews of qualitative evaluations of digital interventions were excluded. Systematic reviews that reported only on intervention design and characteristics with no report on intervention effects were excluded. Only reviews of human studies published in English with the majority conducted in developed countries were included.

#### Types of Outcomes

The primary outcomes were food and nutrition behaviors (eg, dietary intake and eating habits), knowledge (eg, how to read a food label), and attitudes (eg, self-efficacy and intentions). Outcomes related to breastfeeding, weight status (eg, BMI, fat mass, waist circumference, and childhood obesity), health (eg, blood pressure and blood glucose), and nonnutrition topics (eg, physical activity, sedentary behavior, and sleep) were excluded. The secondary outcomes were food and nutrition outcomes according to the behavior change theory and techniques.

### Screening and Quality Appraisal

Titles and abstracts were screened by 3 reviewers with 20% of the results double-screened to ensure high interrater agreement. Full-text articles were retrieved and reviewed by 2 reviewers and confirmed by a third reviewer. Consensus on the included studies was achieved through discussion.

A MeaSurement Tool to Assess systematic Reviews 2 (AMSTAR 2) was used to assess the quality of the systematic reviews [27]. Quality appraisal was completed on all the included articles, with a subsample of reviews completed by 2 independent reviewers to test interrater reliability. No discrepancies in the quality appraisal between the reviewers were identified.

### Data Extraction and Data Synthesis

Relevant information was extracted by 1 author, including study design; methods; population; intervention type; dose; and duration, outcome measurement, results, and limitations. The findings were reviewed and summarized using the systematic review results and conclusions as the primary units of analysis [21]. Where possible, the outcome effect sizes (ESs) were extracted and assessed by intervention type (eg, internet, mobile, and social media) and by outcome type (eg, fruit and vegetable intake). When this was not possible, the overall impact of digital interventions on food and nutrition outcomes was assessed.

## Results

### Study Characteristics

**Figure 1:**
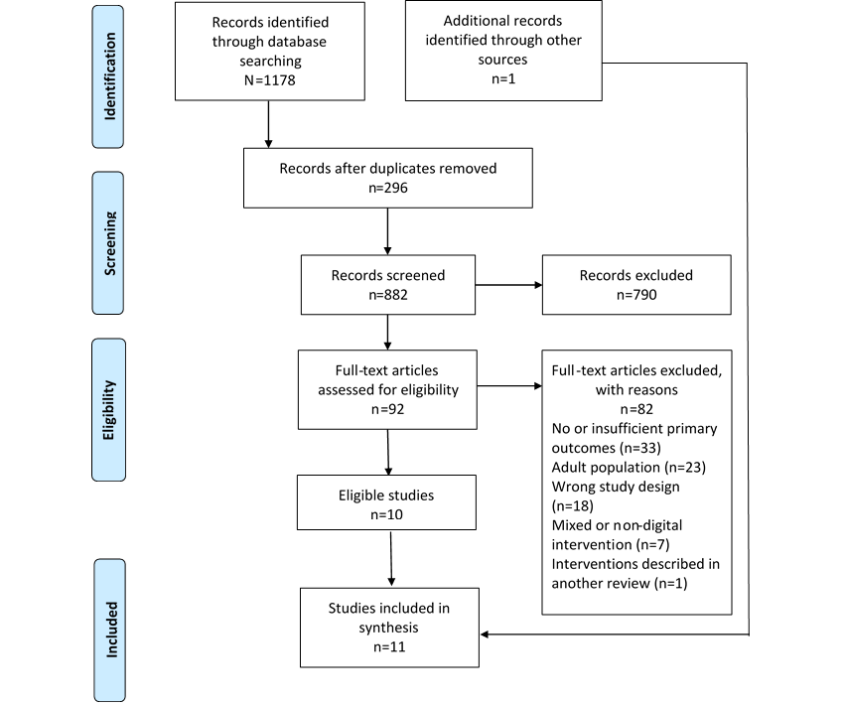
PRISMA (Preferred Reporting for Systematic Reviews and Meta-Analyses) diagram.

The search identified 1178 articles, of which 92 (7.81%) were selected for full-text review, 80 (6.79%) did not meet the inclusion criteria, and 1 (0.08%) was excluded because all interventions were reviewed in a more recent, higher-quality review. As a result, 11 of the 1178 reviews (0.93%) were included to be examined for the impact of digital interventions on nutrition outcomes in children and youth [28-38] (Figure 1). Of the 11 reviews, 3 (27%) included meta-analyses [28,29,32]; 7 (64%) of the reviews were of moderate quality [28-30,33-36], 1 (9%) was of low quality [37], and 3 (27%) were of critically low quality [31,32,38].

The reviews included children between the ages of 7 and 19 years. Of the 11 reviews, 1 (9%) focused on parents of children aged 1 year to early adolescence [30], and 2 (18%) reported separate findings for children and adults [28,31]. These articles were retained because of their quality and unique research focus (single digital modality meta-analyses and behavior change technique (BCT) evaluation [28] and social media [31]).

Interventions ranged from internet, computer, or mobile interventions to websites, programs, apps, emails, videos, CD-ROMs, games, SMS text messages, telehealth, and social media. Most reviews included studies in which the digital intervention was a component of a larger intervention [29-33,36,38], with some including face-to-face components [32,34].

The dose and duration of the digital interventions ranged from a single exposure to multiple sessions (1-60 minutes in length) over 1 or 2 years. Most outcomes were evaluated immediately after the interventions, with few reviews reporting on effects at medium (eg, 2 months) or long (eg, 2 years) follow-ups [29,31,34,35,37]. Interventions were compared with no intervention, nonnutrition digital interventions (eg, websites on physical activity), nondigital nutrition interventions (eg, print healthy eating information and usual nutrition education), and face-to-face interventions, and were often mixed within reviews. Further details on the intervention characteristics can be found in Multimedia Appendices 1 and 2 [28-38].

### Impacts Across All Digital Interventions

In general, reviews have highlighted the promise of the digital interventions to improve diets; however, the evidence of its impact on dietary outcomes in children remains inconclusive. Tallon et al [37] and Wickham and Carbone [38] reported that all studies reported at least 1 positive result in favor of the intervention; however, the findings were mixed when collated across the studies. Do Amaral e Melo et al [33], Zarnowiecki et al [30], and Rose et al [36] also reported a mix of positive, null, and negative impacts of digital interventions across the reviewed studies. Rodriguez Rocha and Kim [28] reported that digital interventions were effective in improving fruit and vegetable intake among adolescents (ES=0.26; SE 0.06; 95% CI 0.14-0.38; *P*<.001) but not among children (ES=0.11; SE 0.11; 95% CI and *P* value were not reported). In studies that evaluated the maintenance of digital intervention effects, positive results from immediate impacts of the interventions were generally not sustained over time [28,29,33,36,37]. Refer Multimedia Appendix 2 for details of the review findings.

### Impact by Digital Modality

#### Internet

Internet-based interventions (eg, websites, social media, or email) were reported in 7 reviews [28-30,34,36,38]. Meta-analyses by Rodriguez Rocha and Kim [28] and Champion et al [29] found small significant impacts of internet-based interventions. Rodriguez Rocha and Kim [28] reported an ES of 0.19 (SE 0.05; 95% CI 0.09-0.29; *P*<.001) on fruit and vegetable intake across 10 internet-based interventions for adults, adolescents, and children (all ages assessed together). Champion et al [29] reported a standard mean difference of 0.11 (95% CI 0.03-0.19; *P*=.007) of digital interventions (14 internet-based; 2 CD-ROMs) delivered in schools on mean servings of fruits and vegetables per day to those aged 11-18 years; however, this effect was not sustained at follow-ups between 2 and 36 weeks. Some positive impacts of the digital interventions (where the majority were internet-based) on fruit and vegetable intake were also reported by Zarnowiecki et al [30] and Hsu et al [34]; however, the results were inconsistent across all studies in these reviews.

Hsu et al [34] also reported mixed results for internet-based interventions on other dietary intake outcomes (eg, SSBs, junk food, and breakfast in those aged 11-18 years from meta-analyses with 3 studies each). Websites (n=7) and apps (n=1) geared toward using parents as agents of change for children’s nutrition were found to have positive impacts on parents’ and children’s knowledge, attitudes, and feeding practices, but had mixed findings on dietary intake [30]. Wickham and Carbone [38] reported mixed findings of digital interventions used for adolescent food literacy programming (7/8, 88% were internet-based) on nutrition knowledge, attitudes (eg, self-efficacy), skills (eg, planning), and intake (eg, fruit and vegetable intake). Finally, Rose et al [36] found that of the 10 website interventions, only 3 (30%) had significant improvements in diet while the remaining 7 (70%) reported null or inconclusive findings.

#### Computer

Tallon et al [37] included 12 computer-based interventions (eg, programs, games, websites, or email) and 1 mobile intervention and found mixed results for nutrition knowledge and dietary changes among those aged 12-18 years.

#### Mobile

From the 3 interventions included in a meta-analysis, Rodriguez Rocha and Kim [28] found that SMS text messaging interventions had a moderate impact on fruit and vegetable intake (ES=0.41; SE 0.1; 95% CI 0.21-0.63; *P*<.01) for adults, adolescents, and children (all ages assessed together). Darling and Sato [32] evaluated mobile interventions (3 SMS text messaging interventions and 4 mobile app interventions) that included self-monitoring of behaviors. This critically low-quality review found a very small effect on fruit and vegetable and SSB intake (assessed together; Cohen *d*=0.10; 95% CI 0.002-0.024) in children with overweight or obesity [32]. Darling and Sato [32] concluded that the true effect of the mobile interventions with self-monitoring was difficult to determine, as few studies were RCTs. Rose et al [36] included only 1 study that evaluated the effect of SMS text messaging on diet and found that there was no impact on fruit and vegetable intake compared with a control condition.

#### Gaming

In a review of 21 digital gaming interventions on nutrition outcomes, most studies reported improvements in nutrition knowledge, eating habits (eg, increased fruits and vegetables, decreased fat, and sugar), and attitudes (eg, intentions, and self-efficacy) [35]. The reported ESs ranged from small to large across a subsample of 6 studies [35]. Rose et al [36] reported on a game-based intervention that found positive impact on fruit and vegetable intake; however, the impacts on other dietary outcomes were unclear. Rodriguez Rocha and Kim [28] assessed gamified interventions on CD-ROMs, mobile apps, and video games, but reported that there was no statistically significant effect on fruit and vegetable intake for all ages. Wickham and Carbone [38] reported mixed findings across all the studies.

#### Social Media

Only 1 critically low review (as per A MeaSurement Tool to Assess systematic Reviews 2) reported that 50% (8/16) of studies found at least 1 positive impact of social media interventions on dietary outcomes (eg, fruit and vegetable intake and SSB intake) [31]; however, it is unclear whether the results were consistent across studies. The authors noted that the social media interventions were highly heterogenous, often with various BCTs and as a component of a multicomponent intervention; thus, the impact of social media itself is difficult to determine [31].

### Impacts by BCT

Six reviews discussed the use of theories or frameworks in primary studies and found that most interventions were informed by some theory or framework. The most commonly mentioned theories were social cognitive theory [28,31,33,34] and the transtheoretical model (stages of change) [28,31,33,34,39]. A variety of BCTs were incorporated into the digital interventions. Rodriguez Rocha and Kim [28] identified 20 unique BCTs used in 19 studies (mean 4; range 1-7). Instruction or education were used by most interventions [28,30,34,36-38]. Other common BCTs were personalized feedback [28-30,34], goal-setting [28-30,34,36], tailoring interventions to individuals [28] and self-monitoring [29,30,32,36].

Rodriguez Rocha and Kim [28] concluded that digital interventions that incorporated 7 or 8 BCTs had larger effects (ES=0.42; SE 0.1; 95% CI 0.21-0.62; *P*<.001) than digital interventions that used fewer techniques to improve fruit and vegetable intake. However, they did not find any difference in the effectiveness of digital interventions on fruit and vegetable intake by the 5 common BCTs: instruction, feedback, goal-setting, identifying barriers, and explaining consequences of behavior. Interventions that were tailored (ES=0.27; SE 0.05; 95% CI 0.16-0.37; *P*<.001) and nontailored (ES=0.22; SE 0.11; 95% CI 0.00-0.44; *P*=.05) were both effective and not significantly different. Rose et al [36] reported that significant improvements in at least one diet outcome were found more often in digital interventions that included goal-setting; digital interventions that included self-monitoring techniques were more effective if they also included goal-setting.

Do Amaral e Melo et al [33] stated that all studies that used the social cognitive theory showed immediate significant positive outcomes but could not conclude that the impacts were due to the use of this theory. Similarly, Champion et al [29] stated that better outcomes were found when interventions were guided by the transtheoretical model and provided personalized feedback to students; however, this was not analyzed in the review.

## Discussion

### Principal Findings

There is substantial evidence on digital nutrition interventions; however, there was significant heterogeneity in the research regarding the types of digital interventions included, intervention duration, follow-up evaluation timing, comparison groups, and dietary outcomes. As a result, the evidence on their effectiveness remains unclear and inconsistent. Although the evidence was limited, the use of BCTs and techniques appeared to be important in increasing the effectiveness of the digital interventions [28,29,33].

The digital nutrition interventions seemed to be the most promising for improving fruit and vegetable intake; however, many reviews have found mixed results. For example, a moderate quality review by Rodriguez Rocha and Kim [28] that focused solely on vegetable and fruit intake found a small overall impact of digital interventions on adolescents but not children. There was limited evidence on the impact of digital interventions on other food literacy outcomes, including nutrient intake, food and nutrition knowledge, attitudes, and skills. The inconsistent and mixed results from the included reviews may be due to the variability in quality, study design, and outcomes measured. In addition, owing to the heterogeneity of the interventions, few reviews performed meta-analyses to estimate the overall ESs.

The observed positive effects of digital interventions on dietary outcomes ranged from small to medium [28,29,32] and were comparable with the ESs of the traditional nutrition interventions for children [40,41]. In a review of nondigital nutrition interventions, less than one-third of the reported ESs were above 0.2 and statistically significant [40]. Another systematic review and meta-analysis of the traditional school-based nutrition education interventions showed small to medium effects (between 0.14 and 0.40) on fruit and vegetable intake, sugar intake, energy intake, and nutrition knowledge [41]. Thus, it is reasonable to expect digital nutrition interventions to generate ESs in the small to medium range. Similarly, digital nutrition interventions appeared to moderately improve dietary outcomes immediately after the intervention but were not well maintained over time. The long-term success of both traditional [40,42,43] and digital [28,29,33,35,44] nutrition interventions have not been well-studied.

It is unclear whether certain types of digital interventions are more effective than others, as most studies were unable to compare individual modalities and many interventions were multicomponent. Multiple digital intervention types have often been assessed collectively in reviews, making it impossible to distill the impacts by the digital modality and separate the effect resulting from digital aspects from other aspects of the intervention [31,37,38]. Even when digital interventions are assessed independently, inconsistency between reviews impedes the evaluation of the strength of evidence. For example, a website may have been counted as an internet-based intervention in 1 review and a computer-based intervention in another; a mobile app may be counted as a mobile-based intervention or a gaming intervention. Other important features of digital nutrition interventions that may be important for effective interventions are personalized feedback, participant interaction with researchers, duration of at least 3 months, and objectives and activities aligned with specific target behaviors [44]. A meta-analysis of mobile apps aimed at improving the diets in children <18 years found that modeling and social support were significant predictors of intervention ES on dietary outcomes (eg, fruit and vegetable intake and nutrient intake); practicing target desirable behaviors (eg, eating vegetables) was a significant predictor of intervention ES for children but not adolescents [45].

Research on adults found that digital engagement using the telephone or SMS text messaging was more effective than other modalities such as websites, which the authors posit may be attributable to the use of direct communication [46]. Similarly, Brigden et al [16] found that children’s direct connections with a health professional during the digital interventions to manage chronic diseases made a difference in its effectiveness on nutrition outcomes for those aged between 5 and 12 years. There are several factors that impact user engagement with technology (eg, personal traits, beliefs, privacy, and technological challenges) [47], which vary widely across interventions included in the reviews; thus further muddying our understanding of the promise of digital interventions. Nonetheless, the pandemic has expanded opportunities to use eHealth interventions for multiple populations (eg, rural communities, lower socioeconomic status, and youth) [20].

Consistent with another review of web-based nutrition interventions [44], the use of behavior change theories and techniques was associated with increased intervention effectiveness [28,29,33]. This may be different from face-to-face interventions; Murimi et al [48] found that the theory-based face-to-face nutrition interventions for children aged between 2 and 19 years did not perform better than those interventions that were not theory-based. Black et al [40] also stated that the theoretical basis of family, school, and childcare nutrition interventions delivered in a conventional format was not associated with their effectiveness. Other factors such as parent engagement, supportive environments and policies, and activities aligned with specific target behaviors may be more important than the use of a theory in the design of childhood nutrition interventions [48]. Furthermore, Duan et al [46] recommended that the digital interventions target multiple levels of the socio-ecological model to generate a greater impact. Owing to the number and variety of determinants of diet, an intervention that targets only 1 level (eg, individual knowledge) may not be expected to generate large impacts [46].

Many questions remain regarding the best practices to implement digital interventions. The evidence reviewed did not yield information on digital accessibility, acceptability, usability by participants, intervention logistics (eg, how to provide food and cooking equipment to participants in a remote cooking program), participant engagement, privacy and security, equity, and cost-effectiveness [36]. Digital accessibility may be particularly important as some populations do not have the means to access technology, and if those with greater access to resources are better able to engage with digital interventions, there is potential for these digital interventions to increase health inequities. Moreover, the *scale-up penalty* of adopting interventions must be considered, as the effects seen in RCTs may not be effective to the same extent in real-life implementation [49]. Nutrition interventions, including digital interventions, should be carefully designed and implemented [40,41] and rigorously evaluated using RCTs, should contribute to a series of supporting interventions for healthy eating [40,48,50], and strive to reduce health and diet inequities.

### Limitations

There are many challenges in conducting umbrella reviews [22]. Our conclusions are limited by the inability to assess the strength of evidence, such as using Grading of Recommendations Assessment, Development and Evaluation, owing to heterogeneity. Weaknesses in the primary studies in the reviews further reduce certainty in the conclusions. Many reviews included studies with nonrandomized or quasi-experimental designs, cross-sectional studies, and pre-post study designs. Reviews often collectively evaluated poorly described heterogenous interventions with various comparison group types and multiple outcomes, which limited our ability to aggregate findings by individual digital intervention type across the reviews. In general, the included studies had very small sample sizes and often used convenience sampling. ESs were rarely published, which limited our ability to draw conclusions about the effectiveness of digital nutrition interventions. These challenges are not unusual; Murimi et al [44] also cited inconsistent comparison groups, lack of intervention details (eg, dosage), lack of tracking participant engagement, subjective outcome measurement, and lack of follow-up as challenges in reviewing the digital nutrition interventions.

The findings of this review are further limited by the speed at which technology advances and the current evidence on digital interventions that may not have sufficiently evaluated the digital modalities that are popular today, such as videoconferencing or social media. In contrast, despite including the most recent reviews on this topic, CD-ROM interventions were evaluated in reviews published in 2019. Nonetheless, the feasibility and effectiveness of the digital interventions is valuable to explore, as they may have benefits regarding population reach or cost-effectiveness [44]. Owing to these limitations, we have been careful not to overstate the promise of digital interventions as the positive findings may have been inflated due to publication bias, overlap between reviews, and research quality.

### Conclusions

The effect of digital interventions on food and nutrition outcomes is small and inconsistent. Nevertheless, digital adaptations or additions to these interventions based on behavior change theory and techniques may be considered, as web-based service delivery has become increasingly common worldwide. Digital technologies provide an opportunity to increase the reach of interventions and reduce costs, resources, and efforts required to produce or deliver programing. High-quality evidence with common definitions for digital intervention types and evaluation with validated measures is needed to improve the state of evidence to inform policy and program decisions for health promotion in children. Now is the time for critical, robust evaluation of the digital interventions adopted during and after the COVID-19 pandemic to establish effective best practices for eHealth nutrition interventions for children.

## Appendix

Multimedia Appendix 1 Characteristics of digital nutrition interventions for children.

Multimedia Appendix 2 Study characteristics and findings of digital nutrition interventions for children.

## Abbreviations

AMSTAR 2: A MeaSurement Tool to Assess systematic Reviews 2
BCT: behavior change technique
ES: effect size
RCT: randomized controlled trial
SMD: standard mean difference
SSB: sugar-sweetened beverage
