# Supplementary material for: Digital Interventions to Promote Healthy Eating in Children: Umbrella Review
Source: JMIR Pediatr Parent. 2021 Nov 25;4(4):e30160. doi: 10.2196/30160 (PMC8663671; doi:10.2196/30160)
Supplement: Multimedia Appendix 1 [file pediatrics_v4i4e30160_app1.doc]

Supplemental Table 1. Characteristics of digital nutrition interventions for children

| Author | Objective | Intervention Type | Intervention Duration | Intervention Dose | Intervention behavior change characteristics | Comparator(s) | Outcomes measured |
| --- | --- | --- | --- | --- | --- | --- | --- |
| **Rodriguez Rocha & Kim, 2019** [28] | Evaluate effectiveness of eHealth interventions on fruit and vegetable intake among healthy population compared to minimal or no intervention | Internet (n=9), Computer (including CD-ROM [n=2], Game [n=1]) (n=6), SMS (n=3), Mobile app (n=1) | Mean: 9.3 weeks Range: 1 session-24 weeks | Not specified | Theory: n=11/19 studies - Social cognitive theory (n=3); Transtheoretical Model (n=3)  Techniques: n=18/19 studies – mean of 4 techniques used (range 1-7). Most common techniques: 'provide instruction on how to perform the behavior' (n=13), 'provide feedback on performance' (n=9), 'goal setting on behavior' (n=8)  Other: Intervention tailored in n=15/19 studies | No intervention (n=10), minimal nutrition non-digital intervention (n=5), minimal non-nutrition intervention (n=4) | Dietary intake: F&V (n=19) |
| **Zarnowiecki et al., 2020** [30] | Review efficacy of websites and apps targeting parents to improve children’s dietary intake and parent’s and children’s self-efficacy | Internet (websites) (n=7), Mobile app (n=1) | Range: 1 session to 7 months | Range: single exposure to 22 sessions, with durations from 2-30 minutes | Not specified. | No intervention (n=3), non-nutrition digital (n=1), nutrition non-digital (n=1), minimal digital nutrition (n=1), face-to-face (n=1), waitlist (n=1) | Dietary intake: F&V (n=8), SSB (n=3), candy/snacks (n=4), eating habits (n=2), general (n=1) |
| **do Amaral e Melo et al., 2017** [33] | Evaluate the quality and effectiveness of technologies used for nutrition interventions for adolescents | Computer games (n=4); Computer program with tailored feedback (n=4), Computer CD-ROM (n=1), Mobile SMS (n=2) | Median: 7 weeks Range: 1 session to 12 months | Range: single exposure to weekly or daily activities, with durations from 15-60 minutes | Theory: 5/11 studies - social cognitive theory (n=4), theory of reasoned action (n=1), theory of planned behavior (n=1), social learning theory (n=2) | No intervention (n=5), nutrition non-digital (n=2), non-nutrition non-digital (n=1), digital non-nutrition (n=1); traditional education (n=2) | Dietary intake: F&V (n=4), SSB/sugar (n=3), fat (n=2), other (n=3)  Nutrition knowledge (n=1)  Nutrition attitudes (e.g., Self-efficacy (n=3) |
| **Hsu et al., 2018** [34] | Determine the effectiveness of social media-based interventions in improving nutrition for adolescents; identify behavior change techniques used by effective interventions | Internet-platforms or websites | Range: 1 month - 2 years | Not specified. | Theory: n=5/7 studies - social cognitive theory (n=2), social learning theory (n=1), transtheoretical model (n=1), attitude, social influence, and self-efficacy model, and a public health promotion model  Techniques: n=7/7 studies – social support (n=7), instructions and demonstration (n=6), personalized dietary feedback (n=6), goal setting (n=5), self-monitoring (n=5), rewards and incentives (n=4) | Minimal nutrition digital (n=4); face-to-face (n=1), traditional education (n=1), no intervention (n=1) | Dietary intake: F&V (n=6); SSB (n=4); fat (n=1), other (n=3), general (n=3) |
| **Mack et al., 2017** [35] | Examine impacts of video games intended to improve nutrition knowledge, healthy eating, eating habit, food attitudes and/or physical activity | Gaming (educational, interactive, and/or tailored computer, online, video, or adver-games) | Median: 5 weeks  Range: 1 day to 2 years | Range: single exposure to weekly or daily activities, with durations from 5-60 minutes | n/a | No intervention (n=4), intervention delay (n=2), other nutrition gaming (n=4), traditional education (n=3), other digital non-nutrition (n=2), non-gaming digital nutrition (n=2), non-digital nutrition (n=1),  not stated (n=3) | Dietary intake: F&V (n=5), SSB/ sugar (n=2), fat (n=1), water (n=1), energy (n=2), eating habits (n=7)  Nutrition knowledge (n=7)  Attitudes (e.g., self-efficacy, intentions) (n=4) |
| **Champion et al., 2019** [29] | Systematically review the effectiveness of eHealth school-based interventions | Internet (n=14), CD-ROM (n=2) | Range: 1 day to 36 months | Range: single exposure to 15 lessons, with durations 15-60 minutes | Theory: n=12/16 studies - transtheoretical model (n=6), theory of planned behavior (n=3)  Techniques:  computer-tailored feedback (n=10), multimedia components (audio, animations) (n=13), goal setting (n=2), self-monitoring (n=1) | no intervention (n=7); regular education (n=4), face-to-face (n=3), other digital (n=1); minimal digital (n=1) | Dietary intake: V&F (n=9); fat (n=3); SSB (n=4) |
| **Rose et al., 2017** [36] | Synthesize evidence on effectiveness of digital interventions to improve diet quality and increase physical activity in adolescents | Websites (n=15), SMS (n=4), Games/Apps (n=3), Email (n=1) Social media (n=1), or Multicomponent with some digital (n=3) | Range: Single session to 1 year weeks | Range: Single exposure to 12 sessions; some weekly or daily, with durations from 15-20 minutes. | Techniques:  Education (n=22), goal-setting (n=11), self-monitoring (n=14) | For diet studies: regular education (n=5), other nutrition digital (n=2), non-nutrition digital (n=2),  No control (n=2), not stated (n=6­) | Dietary intake: F&V (n=4), SSB (n=2), fat (n=2)  general (n=7)  Eating habits (n=3) |
| **Tallon et al., 2019** [37] | Assess the impact of school-based nutrition education programs with technology on nutrition knowledge and behavior change in adolescents | Computer (program, game, website, email) (n=12) with tailored feedback (n=6), Games (n=2), CD-ROM (n=2), Internet learning (n=2), Mobile (SMS) (n=1) | Range: 1 session to multiple sessions | Range: single exposure to weekly to 2x/day exposure, with durations from 2-50 minutes. | Not specified. | no intervention (n=4); regular education (n=4); non-digital nutrition (n=2); minimal nutrition digital (n=2) no control (n=1) | Dietary intake:  F&V (n=4), fat (n=4) intake, other (n=4)  Eating behaviors (n=3)  Nutrition knowledge (n=4) |
| **Darling and Sato, 2017** [32] | Examine the effectiveness of mHealth technologies employing self-monitoring to decrease pediatric weight status and nutrition outcomes | SMS (n=3), App for smartphone or handheld computer (n=4) | Median: 8 weeks Range: 2-20 weeks) | Not specified. | Techniques: self-monitoring (n=7) | Non-digital (n=2), not stated (n=4), no control (n=1) | Dietary intake: F&V (n=5), SSB (n=3) |
| **Chau et al. 2018** [31] | Evaluate the evidence for using social media to promote nutrition among adolescents and young adults | Social media interventions via Smartphone app (n=4), website (n=1), Facebook (n=2), Twitter (n=1), Pinterest (n=1) (n=1), other social networking (n=1), online or discussion forums/blog/journals (n=7) | 6 weeks to 24 months long; 2 month to 24 month follow-up | Not specified. | Theory: 13/16 studies - social cognitive model (n=6), transtheoretical model (n=3), theory of reasoned action (n=1), self-determination theory (n=2), transcontextual model of motivation (n=2), theory of planned behavior (n=3), social network theory (n=1), theory of interactive technology (n=1)  Techniques: 16/16 studies -  communication (n=5), health tracking (n=12), tailoring (n=9), goal setting (n=3), education (n=8), social support (external to social media) (n=6), and gamification (n=4) | Not specified. | Dietary intake: F&V (n=6), SSB (n=5), ‘junk’ (n=3), eating habits (n=2), alcohol (n=1)  Dietary skills (n=2)  Attitudes (e.g. disordered eating, self-efficacy) (n=3) |
| **Wickham & Carbone, 2018** [38] | Examine the impact of adolescent food literacy programs that use technology, on dietary intake | Internet or web-based platforms (n=7) and games (n=6), either sole intervention or part of multi-component. | Range for RCTs: 2-9 weeks  Range for non-controlled interventions: 5 days to 6 months | Range: single exposure to 9 sessions, with durations from 1-40 minutes | Not specified. | No intervention (n=1), regular education (n=1), non-digital nutrition (n=1), other nutrition digital (n=2), non-nutrition digital (n=1), no control (n=2) | Dietary intake: F&V (n=7), water (n=1), other (n=1)  Nutrition knowledge (n=1)  Food skills (e.g. planning) (n=2)  Attitudes (e.g. self-efficacy) (n=3) |

F&V = Fruit and vegetable; SSB = sugar-sweetened beverage; SMS = short message system
