# Supplementary material for: Digital Interventions to Promote Healthy Eating in Children: Umbrella Review
Source: JMIR Pediatr Parent. 2021 Nov 25;4(4):e30160. doi: 10.2196/30160 (PMC8663671; doi:10.2196/30160)
Supplement: Multimedia Appendix 2 [file pediatrics_v4i4e30160_app2.docx]

**Multimedia Appendix 2.** Study characteristics and findings of digital nutrition interventions for children.

| Study | Types of studies included | Population | Intervention | Comparator | Findings | Quality |
| --- | --- | --- | --- | --- | --- | --- |
| Rodriguez Rocha and Kim, 2019 [28] | - N^a^=19 studies - RCT^b^: 14 (74%), quasi-experiment: 5 (26%) - 17 (89%) studies included in meta-analysis | Healthy adults: 11 (58%) studies, adolescents: 4 (21%), children: 4 (21%) | eHealth (digital intervention via mobile, internet, computer, video games); included only interventions where eHealth was prominent component | No intervention: 10 (53%), minimal nutrition non-digital intervention: 5 (26%), minimal non-nutrition intervention: 4 (21%) | - 19 (100%) studies had positive results - 10 (52%) studies had statistically significant *(P<*.05) results - Overall: ES^c^=0.26 (SE 0.05, 95% CI 0.17-0.35; *P*<.001) - Adolescents: ES=0.35 (SE 0.10, 95% CI 0.16-0.55; *P*<.001) - Children: ES=0.13 (SE 0.11, 95% CI and *P* value not given but indicated as not significant) - Digital modality (assessed for all ages together): SMS text message ES=0.41 (SE 0.1, 95% CI 0.21-0.63; *P*<.01) - Internet ES=0.19 (SE 0.05, 95% CI 0.09-0.29, *P*<.001) - CD-ROM, mobile apps, video games did not have statistically significant positive ES. Outcomes did not differ by good, fair, poor quality evidence | Moderate |
| Zarnowiecki et al, 2020 [30] | - 8 studies (11 papers) - RCT: 4 (50%); quasi-experiment: 1 (13%); pre-/post-studies: 3 (38%) | Parents of children aged 1-13 years | Websites and apps targeting parents where >50% of content is nutrition | No intervention: 3 (38%), non-nutrition digital: 1 (13%), nutrition nondigital: 1 (13%), minimal digital nutrition: 1 (13%), face-to-face: 1 (13%), waitlist: 1 (13%) | - 7 (88%) studies found positive effects of digital intervention on child nutrition. Improvements in dietary intake were small to moderate. F&V^d^ increased in 5 (63%) studies (eg, 1.84 cups of F&V at 1- and 2-year follow-up in mothers of children aged 4 to 6 years). - One-third of the studies found positive impacts of digital intervention on SSB^e^ intake. | Moderate |
| do Amaral e Melo et al, 2017 [33] | - 11 studies - RCT: 7 (64%); quasi-experiment: 4 (36%) | Healthy, aged 9-17 years | Information and communication technologies (eg, email, websites, computer programs, smartphones, text messages, games). 9 (82%) studies used computer-mediated interventions (eg, program, website, game, email) | No intervention: 5 (45%), nutrition nondigital: 2 (18%), non-nutrition nondigital: 1 (9%), digital non-nutrition: 1 (9%), traditional education: 2 (18%) | - 45% (5/11) of studies had positive statistically significant effects from baseline and/or comparison. Positive findings for F&V were reported in 83% (5/6) of studies, especially for those with low-intakes at baseline in 2 studies. - 100% (1/1) of the studies reported improved sugar intake and 100% (2/2) of the studies reported improved SSB intake. 100% (1/1) of the studies reported decreased junk food intake. 100% (2/2) of the studies reported that nutrition knowledge and attitudes improved. Immediate benefits were not maintained in 75% (3/4) of the studies that had longer follow-ups (eg, 6 months). | Moderate |
| Hsu et al, 2018 [34] | - 7 studies (14 papers) - RCT: 4 (57%), quasi-experimental: 2 (29%), uncontrolled feasibility study: 1 (14%) | Aged 13-18 years | Internet only programs: 3 (43%) and face-to-face programs supplemented with internet/website: 4 (57%). Main forms of social media were researcher-moderated discussion boards or chat rooms; one study included online interaction among peers | Minimal nutrition digital: 4 (57%), face-to-face: 1) (14%), traditional education: 1 (14%), no intervention: 1 (14%) | - Small to moderate positing changes (mostly in F&V and SSB intake). 83% (5/6) of the studies reported increases in F and/or V intake. 50% (2/4) of the studies found significant reductions in SSB. 100% (1/1) of the studies reported that calcium intake increased. 100% (1/1) of the studies reported decrease in junk food but not fast food. 0% (0/1) of the studies reported increased breakfast consumption. | Moderate |
| Mack et al, 2017 [35] | - 20 of 21 studies included diet - RCT (n=9), quasi-experiment (n=4, 3 controlled), pre-/post (n=6, 4 controlled), observational (n=2) | Aged 7-15 years | Video games dealing with nutrition, physical activity and obesity in children between 7 and 15 years with the intention to increase knowledge about nutrition, healthy eating, eating habits, and food attitudes | No intervention (n=4), intervention delay (n=2), other nutrition gaming (n=4), traditional education (n=3), other digital non-nutrition (n=2), nongaming digital nutrition (n=2), nondigital nutrition (n=1), not stated (n=3) | - 88% (7/8) of studies reported that nutrition knowledge improved. 100% (13/13) of the studies reported that eating habits improved, F&V: 80% (4/5), fat: 100% (1/1), sugar intake: 100% (3/3). 100% (3/3) of the studies reported that attitudes (eg, self-efficacy, intentions) improved. Games has small to large ESs on nutrition knowledge and behaviors from a subsample of 6 studies. | Moderate |
| Champion et al, 2019 [29] | - 63% (10/16) of studies (22 papers) included diet - RCT (n=16) - 11 included in meta-analysis | Aged 11-18 years | Universal programs targeting two or more risk factors (diet, alcohol, smoking, physical activity, sedentary behavior, sleep) delivered primarily via eHealth methods (internet, computers, tablets, mobile, or telehealth). N=9 included face-to-face | No intervention (n=7); regular education (n=4), face-to-face (n=3), other digital (n=1); minimal digital (n=1) | - School-based eHealth interventions led to a small but significant increase in average F&V intake (servings/day) immediately after the intervention (SMD^f^=0.11, 95% CI 0.03-0.19; *P*=.007) but were not sustained at follow-up (n=6 for meta-analysis). No effect on mean fat intake (g/day), SMD=−0.06; 95% CI −0.15 to 0.03; *P*=.16 (n=3 for meta-analysis). No effect on SSB intake or high-energy snack intake (n=3 for MA^g^) after intervention (SMD=−0.02; 95% CI −0.10 to 0.06; *P*=.58) or follow-up (SMD=−0.06; 95% CI −0.15 to 0.03; *P*=.21). | Moderate |
| Rose et al, 2017 [36] | - 65% (17/26) of studies (32 papers) included diet - RCT (n=19), quasi-experiment (n=4), nonrandomized controlled (n=2), uncontrolled feasibility study (n=1) | Aged 10-19 years | Interventions used website, mobile phones, smartphones, email, personal digital assistants, social media, or multicomponent intervention with these | For diet studies: regular education (n=5), other nutrition digital (n=2), non-nutrition digital (n=2), No control (n=2), not stated (n=6) | - Findings suggest some improvements with website interventions; however, results were not sustained overtime. Web sites: 30% (3/10) of the diet studies showed significant improvement, 30% (3/10) showed null impact, 40% (4/10) were inconclusive. Positive results were seen for F&V (75% [6/8] of the studies had significant results, but not at 2-year follow-up), fat (67% [2/3] of the studies), other dietary outcomes (energy, snack intake, breakfast, fast food, *junk* food) were assessed by single studies and had mixed results. SMS text messaging: 0% (0/1) of the studies showed improvement (no impact on F&V). Games: 100% (1/1) of the studies showed positive impacts on F&V, but unclear results for other dietary outcomes. Email: 0% (0/1) of studies showed positive impacts on F&V. Multicomponent with digital: 1 intervention with a smartphone app showed positive impacts on SSB, studied in boys only; another intervention with SMS text messaging did not have significant improvements in diet for girls. | Moderate |
| Tallon et al, 2019 [37] | - 13 studies - Designs not specified | Aged 12-18 years | Information and communication technologies (eg, computer, internet, mobile devices) used for nutrition education via classroom sessions, tailored feedback, or customized videos/games | No intervention: 4 (31%), regular education: 4 (31%), nondigital nutrition: 2 (15%), minimal nutrition digital: 2 (15%), no control: 1 (8%) | - All studies reported at least 1 positive result; results inconsistent and sometimes depended upon engagement (participating in sessions/reading material) or sample (eg, low F&V intake at start). Nutrition knowledge increased (but was no different than control in one study); dietary intake improved on some measures (eg, F&V—for low eaters at baseline; fat—for those that participated more) but not all food types or nutrients | Low |
| Darling and Sato, 2017 [32] | - 7 studies (8 effect sizes included in meta-analysis) - RCT: 6 (86%), pre/post (n=1) | Children aged 5-16 years with overweight and obesity | mHealth via apps for smart phones or handheld devices as part of pediatric obesity interventions | Nondigital: 2 (29%), not stated: 4 (57%), no control: 1 (14%) | - Small significant weighted mean ESs of mHealth with self-monitoring on F&V and SSB intake (*d*=0.10, 95% CI 0.002-0.024). | Critically low |
| Chau et al, 2018 [31] | - 16 studies - RCT: 13 (81%), pre-/poststudies: 2 (13%), parallel non− experimental design: 1 (6%) | Aged 10-25 years | “Social media website, application, or homegrown technology that allows users to communicate or share information with peers” | Not specified | - Positive results across eight studies. From Table 2: F&V: 100% (6/6), SSB: 100% (4/4, results not stated in n=1), *junk* foods: 100% (3/3), reduced alcohol: 100% (1/1), eating behaviors 100% (2/2). Dietary skills improved 100% (1/1, results not stated in n=1). Social support for eating improved in 100% (1/1). Dietary self-efficacy results not clearly reported. | Critically low |
| Wickham and Carbone, 2018 [38] | - 8 studies - RCT: 6 (75%), pre/post (n=2) | Aged 8-16 years | Internet and web-based platforms with gaming aspects: 5 (63%), focused on meal planning: 3 (38%), selecting foods: 7 (88%), preparing food: 3 (38%), promoting healthy eating: 8 (100%) | No intervention: 1 (13%), regular education: 1 (13%), nondigital nutrition: 1 (13%), other nutrition digital: 2 (25%), non-nutrition digital: 1 (13%), no control: 2 (25%) | - All studies found at least 1 positive nutrition findings, but findings were mixed. Nutrition knowledge: increased in both intervention and nondigital control in 100% (1/1) of the studies. Nutrition attitudes (self-efficacy): positive findings in 100% (3/3) of the studies. Nutrition skills (planning): positive findings in 100% (2/2) of the studies. F&V intake increased in intervention relative to control in 57% (4/7) of the studies, nonsignificant or no difference between intervention/control (eg, both increased) in 43% (3/7) of the studies. | Critically low |

^a^Total number of studies.

^b^RCT: randomized controlled trial.

^c^ES: effect size.
^d^F&V: fruit and vegetable.
^e^SSB: sugar-sweetened beverage.

^f^SMD: standard mean difference.

^g^MA: meta-analysis
